# A CAR-T response prediction model for r/r B-NHL patients based on a T cell subset nomogram

**DOI:** 10.1007/s00262-023-03618-w

**Published:** 2024-01-27

**Authors:** Xiaomei Zhang, Rui Sun, Meng Zhang, Yifan Zhao, Xinping Cao, Ruiting Guo, Yi Zhang, Xingzhong Liu, Cuicui Lyu, Mingfeng Zhao

**Affiliations:** 1 School of Medicine, Nankai University, Tianjin, China; 2 Department of Hematology, Tianjin First Central Hospital, School of Medicine, Nankai University, No. 2, West Baoshan Road, Xiqing District, Tianjin, 300392 China; 3 State Key Laboratory of Medicinal Chemical Biology, Key Laboratory of Molecular Microbiology and Technology of the Ministry of Education, Department of Microbiology, College of Life Sciences, Nankai University, Tianjin, China; 4 First Central Clinical College, Tianjin Medical University, Tianjin, China

**Keywords:** Refractory/relapsed B cell non-Hodgkin’s lymphoma, Chimeric antigen receptor T cells, Predictive models, Treatment response

## Abstract

**Background:**

Chimeric antigen receptor (CAR) T cells for refractory or relapsed (r/r) B cell no-Hodgkin lymphoma (NHL) patients have shown promising clinical effectiveness. However, the factors impacting the clinical response of CAR-T therapy have not been fully elucidated. We here investigate the independent influencing factors of the efficacy of CD19 CAR-T cell infusion in the treatment of r/r B-NHL and to establish an early prediction model.

**Methods:**

A total of 43 r/r B-NHL patients were enrolled in this retrospective study. The patients’ general data were recorded, and the primary endpoint is the patients’ treatment response. The independent factors of complete remission (CR) and partial remission (PR) were investigated by univariate and binary logistic regression analysis, and the prediction model of the probability of CR was constructed according to the determined independent factors. Receiver operating characteristic (ROC) and calibration plot were used to assess the discrimination and calibration of the established model. Furthermore, we collected 15 participators to validate the model.

**Results:**

Univariate analysis and binary logistic regression analysis of 43 patients showed that the ratio of central memory T cell (Tcm) and naïve T cell (Tn) in cytotoxic T cells (Tc) was an independent risk factor for response to CD19 CAR-T cell therapy in r/r B-NHL. On this basis, the area under the curve (AUC) of Tcm in the Tc and Tn in the Tc nomogram model was 0.914 (95%CI 0.832–0.996), the sensitivity was 83%, and the specificity was 74.2%, which had excellent predictive value. We did not found the difference of the progression-free survival (PFS).

**Conclusions:**

The ratio of Tcm and Tn in Tc was found to be able to predict the treatment response of CD19 CAR-T cells in r/r B-NHL. We have established a nomogram model for the assessment of the CD19 CAR-T therapy response presented high specificity and sensitivity.

**Supplementary Information:**

The online version contains supplementary material available at 10.1007/s00262-023-03618-w.

## Implications for practice

A nomogram was developed to predict the therapeutic response of the CD19 CAR-T infusion product to lymphoma. Based on the receiver operating characteristic curves and the calibration curves, this nomogram identifies several variables with great predictive power for CD19 CAR-T infusion product responses to lymphoma, which provide the basis for clinical adjustment of the treatment plan.

## Introduction

Chimeric antigen receptor T cell (CAR-T) therapy is different from conventional small molecule or biological therapy. It collects T cells from patients’ blood through single cell collection. It can express the chimeric antigen receptor (CAR) to recognize and attack tumor cells expressing specific antigens and after modification. At present, its efficacy in diffuse large B cell lymphoma (DLBCL) has been confirmed [1]. The US Food and Drug Administration (FDA) had approved three CD19 CAR-T products, axicabtagene ciloleucel, tisagenlecleucel and lisocabtagene maraleucel to treat refractory/relapsed B cell non-Hodgkin’s lymphoma (r/r B-NHL). Research has shown that the total effective rate (ORR) of treatment is 52 to 82%, and the complete effective rate (CR) is 40 to 58% [2, 3]. However, some patients are not sensitive to CAR-T therapy, and for those who are effective with CAR-T therapy, studies have found that 50% of patients experience recurrence eventually [4]. This is partly due to the heterogeneous influence of functional T cells, tumor microenvironment [5] and patient baseline status. In addition, early clinical response evaluation is also important to improve the cost-effectiveness due to the high cost of CAR-T therapy. Therefore, an early prediction model of treatment response is crucial for patients.

The factors that influence the clinical response to CAR-T therapy are not fully understood. Several studies have attempted to determine the impact of these clinical factors [5–8], such as the negative correlation between macrophage infiltration and remission status in the tumor microenvironment [5], baseline eosinophil depletion reducing the antitumor efficacy of CAR-T cells [6], and the number of white blood cells (WBC), TP53 mutation, bone marrow blasts as well as CAR-T generation predicting the treatment response estimation of CAR-T therapy [9]. For example, high tumor burden during infusion is associated with poor efficacy of CAR-T cell therapy, indicating that disease control before CAR-T cell administration is important for a better response [8, 10]. Some genetic changes, such as TP53 mutations, have been reported as intrinsic biomarkers of tumors and can inform poor responses [11]. Meanwhile, the efficacy of CAR-T not only depends on tumor factors, but also is influenced by CAR-T cell parameters [12]. If the therapeutic effect of CAR-T cells can be predicted as early as possible and the treatment strategy can be optimized during the treatment, its therapeutic effect can be maximized. Therefore, evaluating the clinical biomarkers of CAR-T therapy is an urgent issue that needs to be explored.

Here, we analyzed patients with B-NHL who received CD19 CAR-T therapy at our center and screened out independent factors of CAR-T treatment response (ChiCTR-ONC-16008911), found that the proportion of baseline T cell subsets collected before infusion was positively correlated with patients’ early treatment response and developed a simple and easy-to-use model to predict the clinical outcome of these patients for the first time.

## Methods

### Patients and study design

A total of 102 patients with refractory and relapsed B-NHL who received CD19 CAR-T therapy at Tianjin First Central Hospital from September 2019 to September 2022 were retrospectively collected. The main inclusion criteria were as follows: (1) B-NHL was diagnosed by histology and immunohistochemistry according to the 2008 WHO classification of hematological malignancies; (2) refractory or relapsed disease according to the National Comprehensive Cancer Network guidelines; (3) autologous T cells were selected to prepare CAR-T products; (4) medical records within 1 month of CAR-T treatment were complete and accessible. Of these, 59 patients were excluded due to incomplete data or due to severe infection during CAR-T infusion. A total of 43 patients with r/r NHL were included in the cohort. Another 15 patients from October 2022 to October 2023 were taken as the validation cohort. Clinical data included clinical characteristics and baseline hematological parameters of each subject, and information on the CAR-T cells used at each infusion was collected. ALL data were accurately extracted from the patient’s electronic medical record system.

**Fig. 1:**
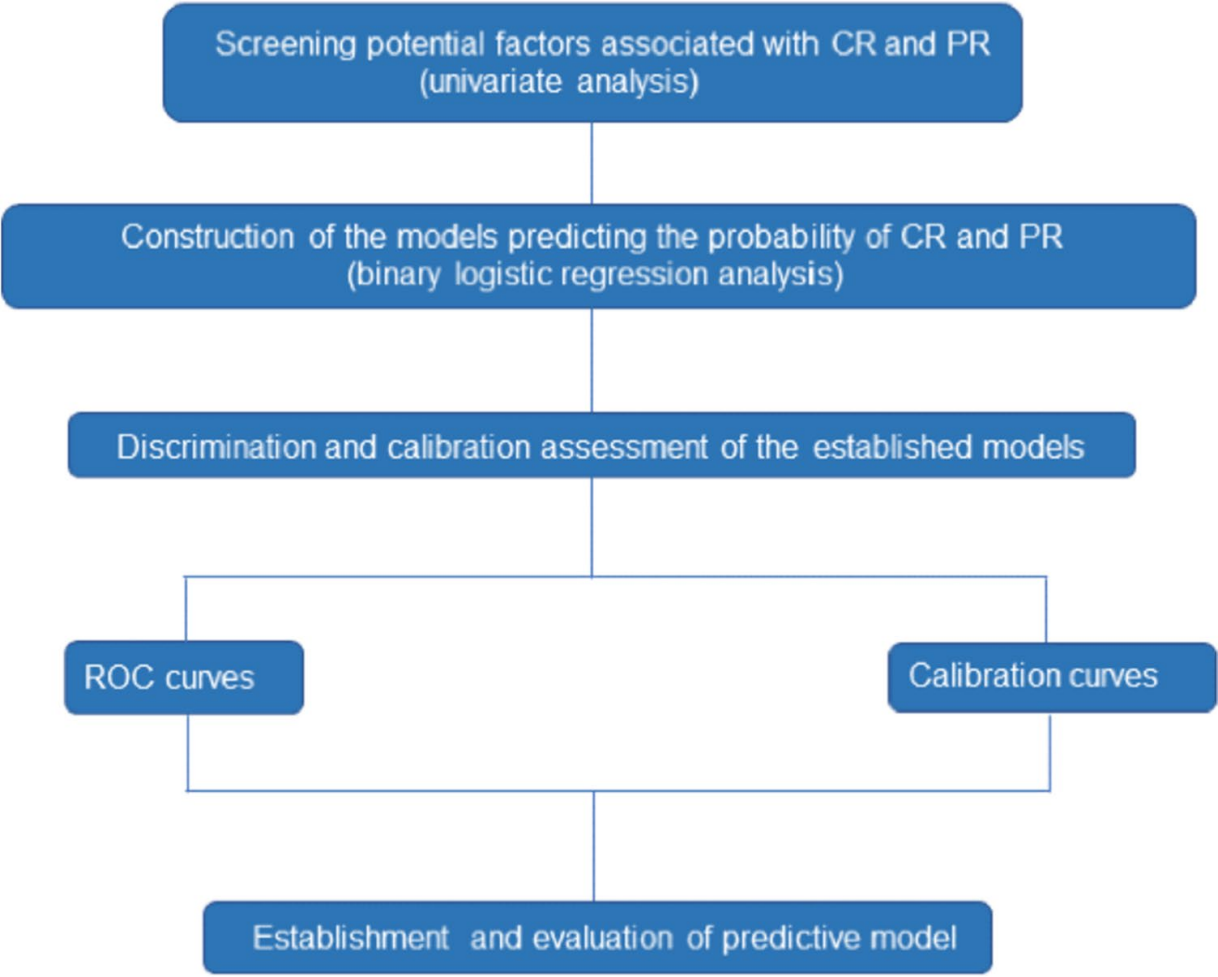
Flowchart of the analytic process of this study

The overall devise of the study is shown in Fig. 1. Baseline characteristics and hematologic parameters were collected for all 43 patients of training cohort and 15 patients of validation cohort, and peripheral blood T cells were tested for T cell subsets, as shown in Table 1. Univariate Mann–Whitney test and binary logistic regression analysis were then used to screen and determine the factors associated with CR and PR in lymphoma patients after CAR-T therapy, including age, gender, baseline tumor burden, bone marrow involvement, previous lines of treatment, ECOG score, baseline T cell subset proportions and CAR-T cell dose. In order to determine the independent risk factors affecting CAR-T efficacy, binary logistic regression was used to perform multivariate analysis and calculate the odds ratio and 95% confidence interval. Subsequently, GraphPad software was used to draw forest maps with risk factors derived from multivariate analysis. At the same time, the prediction model was generated by odds ratio, and then the receiver operating characteristic curve (ROC) curve and the corresponding area under the curve (AUC) were used to evaluate the discriminative ability of the model. Calibration curves were used to estimate the agreement between actual observations and model predictions. The Hosmer–Lemeshow Chi-squared test (c2) was also performed to assess accuracy and goodness of fit. The R software was used to construct a nomogram to obtain a predictive model influencing the efficacy of CAR-T treatment, and the calibration curve was drawn to assess the authenticity and accuracy of the model. Then the ROC curve was drawn to calculate the specificity, sensitivity, Youden index and kappa value to evaluate the prediction model of the nomogram. Furthermore, we also followed up the 58 participants for their survival status. The PFS after CAR-T therapy were compared between different ratio of T cell subset, respectively, to confirm the prognostic value of the prediction model.

**Table 1:** Patient covariates (*N* = 43)

Covariates	n*
Age, median (range)	56 (26–85)
Gender, *n* (%)	
Male	31 (72.1)*
Female	12 (27.9)*
Disease	
DLBCL	36 (83.7)*
MCL	2 (4.7)*
FL	2 (4.7)*
B-LBL	2 (4.7)*
Burkitt	1 (2.2)*
Tumor burden prior CAR-T	
Low	16 (37.2)*
High	27 (62.7)*
Ann Arbor stage	
I-II	8 (15.4)*
III-IV	35 (82.0)*
ECOG score	
< 2	19 (44.2)*
≥ 2	24 (55.8)*
Lines of prior therapies, median (range)	3 (1–7)
Bone marrow infiltration	
Yes	10 (23.3)*
No	33 (76.7)*
Baseline blood count, median (range)	
LDH	225.3 (116.4–7477.8)
CRP	4.52 (0.54–341.19)
Ferritin	379 (34.3–2222)
WBC (× 10^9/L)	3.97 (1.48–13.85)
Hemoglobin (g/dL)	109 (59–145)
Platelet (× 10^9/L)	154 (23–670)
Baseline T cell subset proportions, median (range)	
CD4/CD8	1.1 (0.15–13.43)
Treg (%)	5.71 (0.07–31.47)
Tcm in Th (%)	7.68 (0.38–62.95)
Tcm in Tc (%)	31.88 (3.17–64.77)
Tn in Th (%)	13.55 (1.15–46.77)
Tn in Tc (%)	8.64 (0–48.68)
Teff in Th (%)	2.88 (0.16–50.38)
Teff in Tc (%)	33.43 (0–79.28)
Tem in Th (%)	34.11 (5.72–92)
Tem in Tc (%)	35.12 (0–84.97)
CAR-T cell dose (× 10^6/kg)	3.82 (0.85–12.79)
Outcome variable	
CR/PR	31 (72.1)*
No response	12 (27.9)*

( ) *percentage; *DLBCL* diffuse large B cell lymphoma; *MCL* mantle cell lymphoma; *FL* follicular lymphoma; *B-LBL* B cell lymphoblastic lymphoma; *ECOG* Eastern Cooperative Oncology Group; *LDH* lactate dehydrogenase; *CRP* C-reaction protein; *WBC* white blood cells

### CAR-T therapy

The CAR-T cell product was an investigational product manufactured at our center. The CAR-T cell construct was a CD19-targeting scFv from the FMC63 clone that was combined with human 4-1BB and CD3 signaling domains and loaded into the pCDH-MSCV-MCS-EF1-T2A-Puro vector. The vector was then made ready for transduction with a certain amount of lentivirus. A certain number of mononuclear cells were isolated from the blood of cancer patients using a peripheral blood cell separator, and CD3-positive T cells were sorted by CD3-coated magnetic beads (Miltenyi, 130-097-043). CD3 and CD28-coated magnetic beads (Sigma,11161-D) and interleukin-2 (IL-2, T&L Biotechnology Co., Ltd) were added to stimulate T cell expansion, followed by CAR lentiviral transduction, with a multiplicity of infection (MOI) of 5, and infused into patients after 7–10 days of culture. Lymphodepleting chemotherapy with fludarabine and cyclophosphamide was administered for an average of 3 days prior to the CAR-T infusion. The specific doses were 25 mg/m2/day and 250 mg/m2/day.

### Risk factors and early prediction model of CD19 CAR-T therapy for r/rNHL

**Fig. 2:**
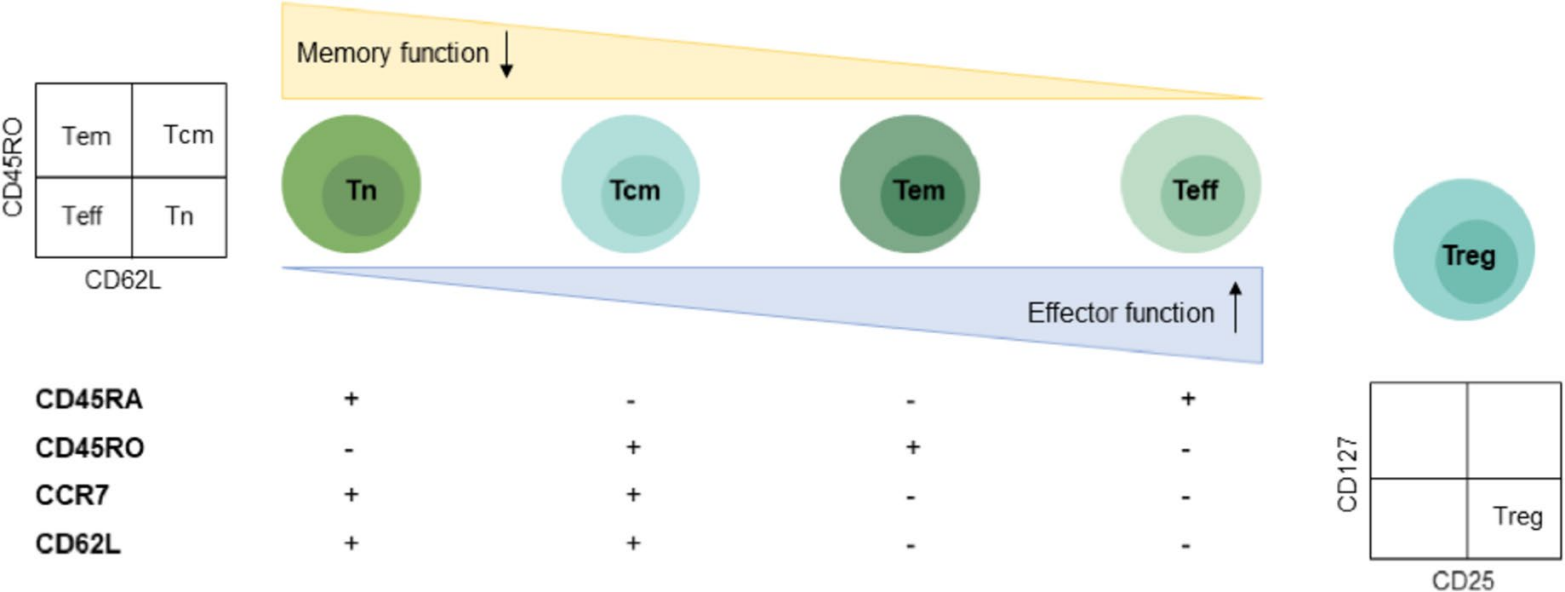
T subgroup flow clustering. Tn, CD45RACD45ROCCR7CD62L^+/−/+/+^; Tcm, CD45RACD45ROCCR7CD62L^−/+/+/+^; Tem, CD45RACD45ROCCR7CD62L^−/+/−/−^; Teff, CD45RACD45ROCCR7CD62L^+/−/−/−^; Treg, CD25CD127^+/−^

Initially, patients’ age, gender, disease type, number of prior lines of treatment and bone marrow accumulation were recorded. Patients’ baseline tumor load was determined by fluorodeoxyglucose positron emission computed tomography (FDG-PET/CT) imaging, and SUVmax < 10 was defined as low tumor load. On the day of apheresis, peripheral blood was collected and sent to the laboratory for a blood count (WBC, hemoglobin and platelets) and blood indices of LDH, CRP and ferritin. T cell subsets can be detected at the same time. After identification by CD3 labeling, T cells were classified as CD4 + Th cells and CD8 + Tc cells according to the expression of CD4 and CD8 on the cell surface. The different subsets of T cell differentiation are shown in Fig. 2, naïve T cells differentiate into central memory T cells, Tcm; effector memory T cells, Tem; and effector T cells, Teff. Different antibody markers in our laboratory (anti-human CD3 Percp, anti-human CD4 FITC, anti-human CD8 Percp-cy7, anti-human CD45RO PE-Vio770, anti-human CD62L APC, anti-human CD127 PE, anti-human CD25 APC, BD Company) were used to identify different markers during differentiation [13]. These tests are routinely performed for the clinical evaluation of patients on the day of collection. Samples were collected on the CytoFLEX flow cytometer, and analyses were performed using CytExpert software (v2.4).

### Response assessment and follow-up

In total, 58 patients were evaluated 1 month after CD19 CAR-T therapy and according to the Lugano efficacy evaluation criteria, including: (1) CR: Imaging showed complete metabolic response of the lesions, Deauville score ≤ 3, no new lesions and no lesions with positive FDG uptake in the bone marrow. (2) PR: Imaging results showed partial metabolic response in the lesion, FDG uptake decreased significantly from baseline with a Deauville score of 4 or 5, no new lesions, FDG uptake decreased from baseline but higher than in the bone marrow. (3) Stable disease (SD): Imaging results showed no metabolic response, Deauville score of 4 or 5 and no significant change from baseline, no new lesions, bone marrow FDG uptake did not change from baseline. (4) Progressive disease (PD): Imaging results showed Deauville 4 or 5 score and FDG uptake increased from baseline, new FDG uptake lesions clearly identified as lymphoma lesions and new or recurrent bone marrow FDG uptake lesions. The last follow-up of the long-term survival of patients from discovery group was on November 5, 2023. PFS was calculated from the date of first infusion to the date of disease progression or death from any cause.

### Statistical analysis

Statistical software SPSS 22.0 (SPSS Inc, Chicago, IL, USA) was used for univariate and binary logistic regression analysis, and graphing software GraphPad Prism 8 was used for statistical analysis and mapping of data. The Mann–Whitney test was used for comparison between samples. Binary logistic regression analysis was performed using the Hosmer–Lemeshow test. For each infusion, patients can obtain the probability score of CR and PR generated by the constructed model, and then the clinical outcome of each infusion is evaluated according to the cutoff value of the ROC curve when the Youden index reaches the maximum value. The R software used was R (4.2.2), RStudio (version 1.4.1717) and the following R packages: rms, readxl and nomogram formula to perform the nomogram in order to obtain a predictive model that influenced the treatment effect of lymphoma patients after CD19 CAR-T treatment, and the calibration curve was drawn to evaluate the authenticity and accuracy of the model. The ROC curve was then drawn, and the specificity, sensitivity, Youden index and other evaluation indicators were calculated. The consistency and reliability of the model prediction were assessed by calculating the kappa value. All reported *P* values are two-tailed, and a *P* value of less than 0.05 was considered significant.

## Results

### Clinical characteristics of the study population

Table 1 shows the patients’ baseline characteristics and the CD19 CAR-T cells they initially received. A total of 43 patients with r/r NHL were enrolled in training cohort, comprising 36 (83.7%) cases of diffuse large B cell lymphoma, 2 (4.7%) cases of mantle cell lymphoma, 2 (4.7%) cases of follicular lymphoma, 2 (4.7%) cases of B cell lymphoblastic lymphoma and 1 (2.2%) case of Burkitt’s lymphoma. There were approximately three times as many men as women, and the median age was 56 years (range 26–85 years). Moreover, ten patients (23.3%) had bone marrow involvement prior to CAR-T cell infusion, and 23 (62.7%) patients had a high tumor burden. Meanwhile, the median number of lines of therapy prior to CAR-T cell infusion was 3 (range, 1–7).

The median dose of infused CAR-T cells was 3.82 × 10^6/kg (range, 0.85–12.79). Meanwhile, the median WBC, hemoglobin, platelet, LDH, CRP and ferritin counts before lymphodepletion (baseline blood count) were 3.97 × 10^9/L, 109 g/L, 154 × 10^9/L, 225.3U/L, 4.52 mg/L and 379 ng/ml. Before treatment, the median CD4/CD8 ratio of peripheral blood T cells was 1.1 (0.15–13.43), the median proportion of Treg cells was 5.71% (0.07–31.47), the proportion of Tcm in Th and Tc cells was 7.68% (0.38–62.95) and 31.88% (3.17–64.77), respectively. The proportion of Tn in Th and Tc cells was 13.55% (1.15–46.77) and 8.64% (0–48.68), respectively. The proportion of Teff in Th and Tc was 2.88% (0.16–50.38) and 33.43% (0–79.28), respectively. The proportion of Tem in Th and Tc cells was 34.11% (5.72–92) and 35.12% (0–84.97), respectively. Thirty-one (72.1%) patients achieved CR or PR one to three months after CAR-T cell infusion. Additionally, the patients characteristics of validation cohort are also presented in Table 1, including 14 DLBCL and 1 FL. To further explore the prognostic value, the long-term survival was compared between the ratio of Tcm in Tc and Tn in Tc. The median follow-up of this study was 9 months (1.37–74.83). In the training cohort, the difference between the proportion of Tcm in Tc (log rank *p* = 0.88) and Tn in Tc (log rank *p* = 0.96) does not cause the comparison difference of PFS (Supplementary Fig. 1).

### Factors associated with CR and PR in r/r NHL patients after CAR-T therapy

**Fig. 3:**
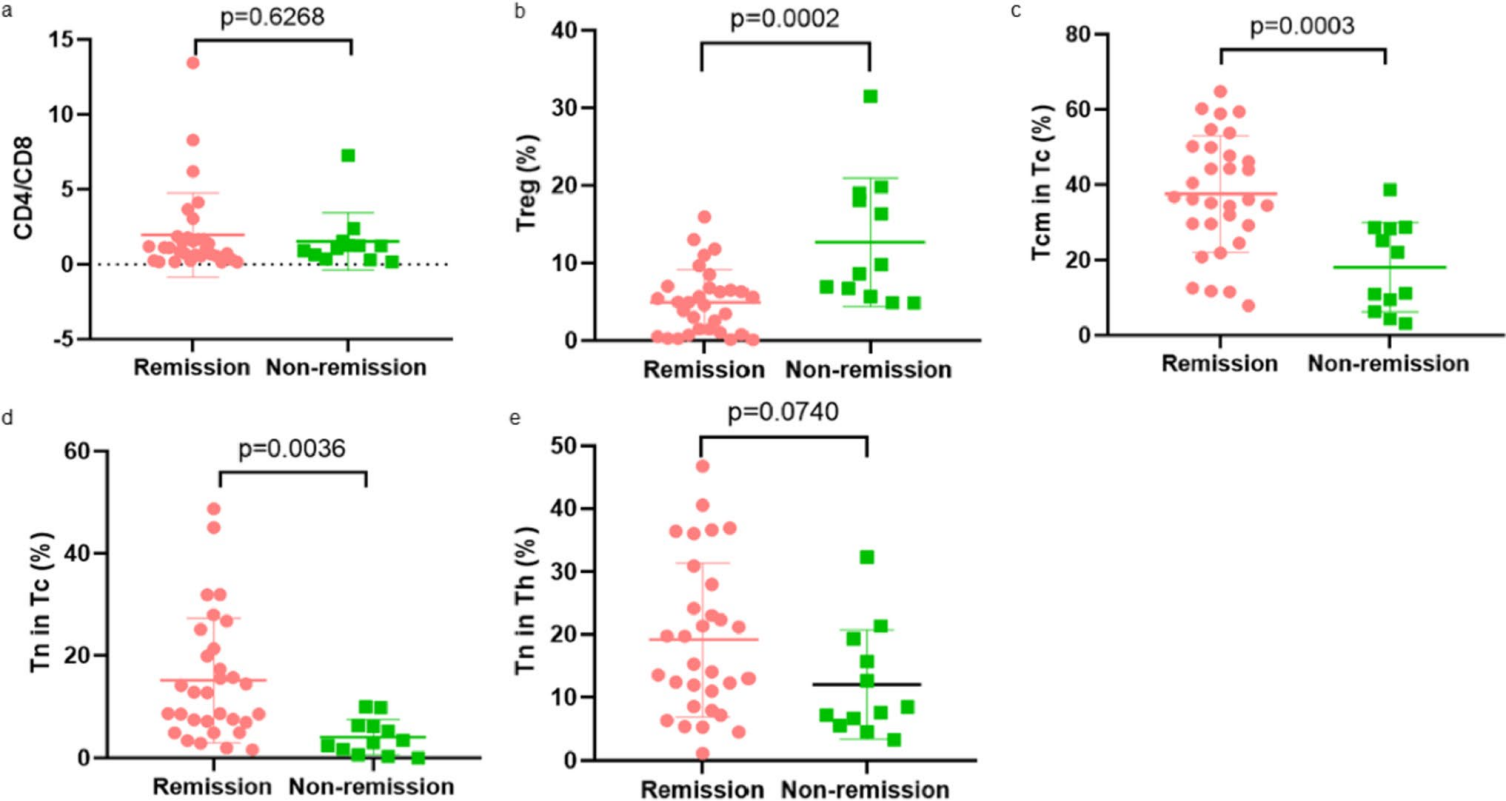
The proportions of T cell subsets in NHL patients. **A** There was no significant difference in the proportion of CD4 + and CD8 + T cells between response and non-response group (*p* = 0.6268). **B** The proportion of Treg cells in the response group was significantly lower than in the non-response group (*p* < 0.05). **C**, **D** The proportion of Tcm and Tn in Tc cells was significantly higher than that in the non-response group (*p* < 0.05). **E** The proportion of Tn in the response group was higher than that in the non-response group, but the difference was not statistically significant (*p* > 0.05)

To search for possible factors for CD19 CAR-T treatment in r/r NHL patients, we first performed univariate logistic regression analysis on patients’ clinical characteristics (Table 2), baseline hematological parameters (Table 3) and the first infusion of CAR-T cells (Supplementary Table 2). The results of univariate analysis showed that the patient’s baseline tumor burden, ECOG score, the proportion of Treg cells in peripheral blood T cell subsets and the proportion of Tcm and Tn in Tc cells at diagnosis were significantly associated with remission (CR and PR) after CAR-T cell infusion (*p* < 0.05). As demonstrated in Fig. 3B–D, the proportion of Treg cells in the response group was significantly lower than in the non-response group (SD and PD). On the contrary, the proportion of Tcm and Tn in Tc cells was significantly higher than that in the non-response group. Although age and the proportion of Tn in Th cells shown in Fig. 3E present correlation with remission, the difference was not statistically significant. However, in Fig. 3A, the CD4/CD8 ratio was not different between the two groups, which may be related to the small sample size.

**Table 2:** Univariate logistic regression analyses of the clinical characteristics of r/r NHL patients associated with CR or PR

Variables	Category	Remission*	No-remission*	*Z*	*P*
Age (years)		60 (46, 68)^#^	46.5 (41, 58.5)^#^	− 1.818	0.071
Gender	Male	22 (70.97)	9 (75)	− 0.261	0.841
	Female	9 (29.03)	3 (25)		
Tumor burden	High	16 (51.61)	1 (8.33)	− 2.409	0.043
	Low	15 (48.39)	11 (91.7)		
ECOG	< 2	18 (58.06)	1 (8.33)	− 3.396	0.002
	≥ 2	13 (41.94)	11 (91.7)		
Bone marrow infiltration	Yes	6 (19.35)	4 (33.33)	− 0.962	0.495
	No	25 (80.65)	8 (66.67)		
Lines of prior therapies	< 4	19 (61.29)	3 (25)	− 1.422	0.174
	≥ 4	12 (38.71)	9 (75)		

() ^#^Interquartile range, percentage for other covariates; *remission includes CR and PR, no-remission refers to NR

**Table 3:** Univariate logistic regression analyses of the blood index of r/r NHL patients associated with CR or PR

Variables	Remission*	No-remission*	*Z*	*P*
WBC	3.53 (2.57, 5.93)	5 (3.625, 6.39)	− 1.476	0.142
Hemoglobin	111 (95, 126)	108 (90.5, 118)	− 0.555	0.584
Platelet	153 (118, 212)	184.5 (141.25, 284.75)	− 1.489	0.142
CRP	4.52 (1.12, 26.04)	6.57 (1.135, 40.7025)	− 0.108	0.926
Ferritin	321 (196, 535)	551 (289, 692.3)	− 1.543	0.127
LDH	217.2 (191, 334.4)	334.05 (231.575, 484.925)	− 1.462	0.149

( ) Percentage; *remission includes CR and PR, no-remission refers to NR

### Identification of independent factors influencing response to CAR-T therapy

**Fig. 4:**
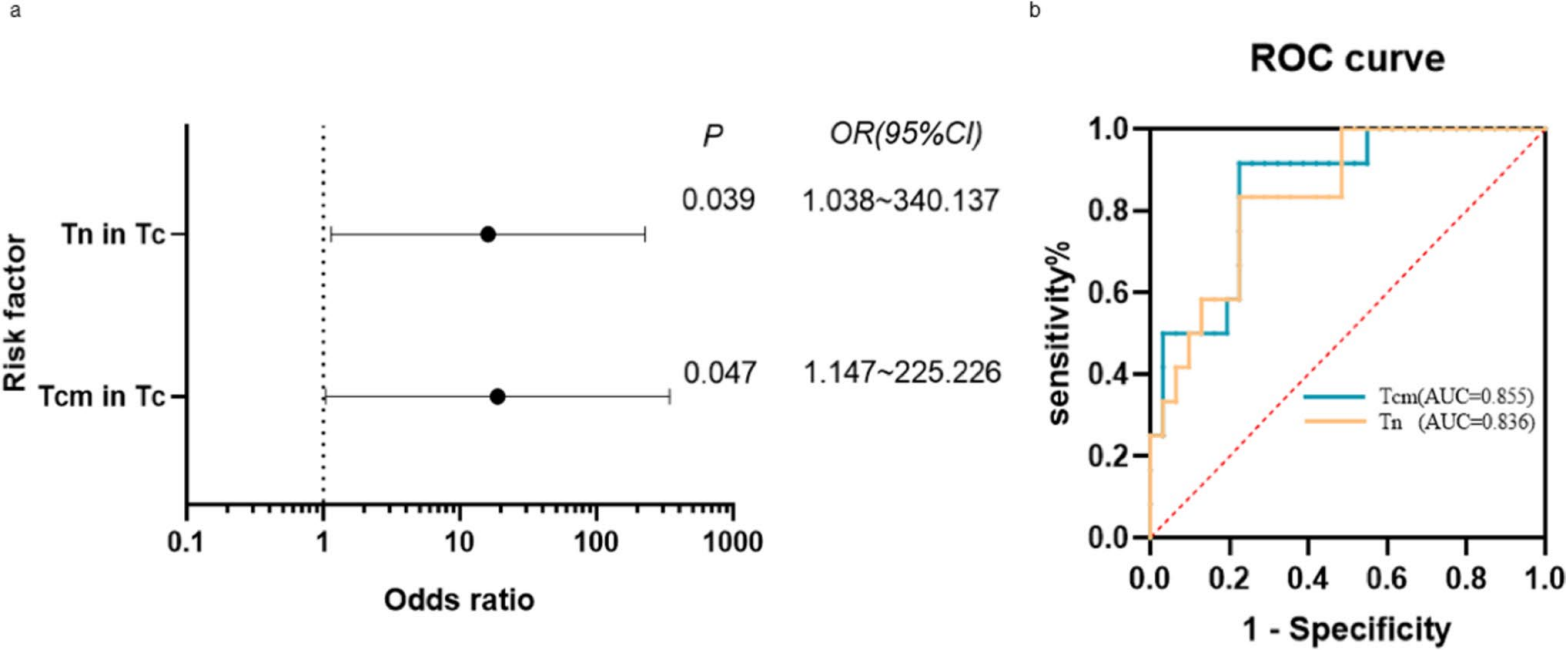
Discriminative ability of Tcm and Tn in Tc between CR and NR. **A** Multivariate logistic regression analysis showed that Tcm and Tn in Tc were closely related to CAR-T treatment response (*p* < 0.05). **B** The ROC curve showed the ability of the T subsets to discriminate between the response and non-response groups. Tcm and Tn in Tc represented good discriminative power with AUC > 0.6, respectively

To find out more about the independent factors for response to CD19 CAR-T therapy, the statistically significant factors from the above univariate analysis (univariate logistic *p* 0.05) were added to the binary logistic regression analysis (Supplementary Table 3). The results of the binary analysis showed that the changes in the proportions of T cell subsets collected from the patients’ peripheral blood before CD19 CAR-T infusion were significant independent factors for the therapeutic effect of CAR-T cells, among which the proportions of Tcm and Tn in Tc cells significantly influenced remission (*p* < 0.05) (Fig. 4A). ROC curve results showed that the AUC of Tcm in Tc and Tn in Tc for predicting the efficacy of CAR-T cell therapy was > 60%, the AUC of Tcm in Tc was 0.855 (95%CI 0.737–0.9730), and the AUC of Tn in Tc was 0.836 (95%CI 0.712–0.960) (Supplementary Table 4) (Fig. 4B).

### Establishment and validation of an early prediction model for CD19 CAR-T treatment response

**Fig. 5:**
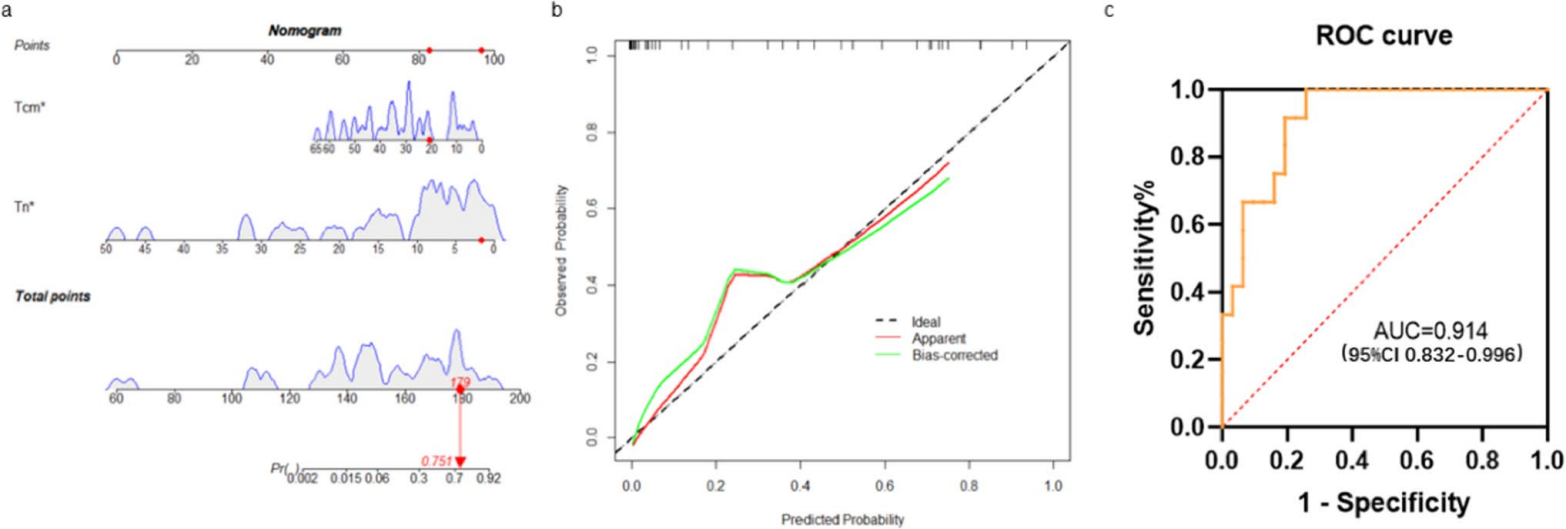
The early prediction model nomogram and calibration curve. **A** A nomogram was created based on the proportion of Tcm and Tn in Tc to predict response to CAR-T treatment. **B** Calibration curves of the early predictive model comparing the predicted probability of response. **C** The total scores of Tcm and Tn were calculated by the nomogram prediction model, and the ROC curve was drawn to predict the response to CAR-T therapy. AUC = 0.914 (95%CI 0.832–0.996)

Potential biomarkers with concurrent AUC > 0.6 were identified based on the selection criteria of *p* < 0.05 between the remission and non-remission groups. We therefore selected Tcm in Tc cells and Tn in Tc cells as predictive biomarkers of early response after CD19 CAR-T therapy. Based on the baseline percentage of Tcm and Tn in Tc cells, we developed an early prediction model of the treatment effect to analyze the likelihood of remission. Then, to assess remission response in clinical practice, we developed a nomogram that could help predict the likelihood of CR and PR for each individual in clinical practice. Finally, we internally validated the original data and drew a calibration curve to further evaluate the authenticity and accuracy of the prediction model in the actual situation. The results showed good agreement between the likelihood of predicting remission and the observed early response (Figs. 5A and B). The total scores of Tcm and Tn were calculated in the nomogram, and the ROC curve was drawn to predict the treatment effect. The AUC result was 0.914 (95%CI 0.832–0.996), showing good predictive ability (Fig. 5C). Moreover, we further validated this predictive model in an independent cohort. The internal validation cohort consisted of 15 r/r B-NHL patients enrolled during a subsequent period of time. The baseline characteristics of the validation group are shown in Supplementary Table 1. According to the established predictive models for response, ROC curve results of validation cohort showed that the AUC of Tcm in Tc and Tn in Tc for predicting the efficacy of CAR-T cell therapy was > 60%, the AUC of Tcm in Tc was 0.860 (95%CI 0.6587–1.0000), and the AUC of Tn in Tc was 0.940 (95%CI 0.8137–1.0000) (Supplementary Fig. 2A). The ROC curve was drawn to predict the validation cohort treatment effect, and AUC result was 0.9301 (95%CI 0.8568–1.0), showing good predictive ability (Supplementary Fig. 2B). The results showed that the prediction model had high sensitivity and specificity in predicting the effect of CD19 CAR-T treatment (sensitivity 83%, specificity 74.2%), and the maximum Youden index was 0.742, and the prediction results were in good agreement with the actual situation (kappa, 0.616), indicating that the model has good predictive ability and has some clinical application value.

## Discussion

Chimeric antigen receptor-modified T (CAR-T) cells have shown significant advantages in the treatment of hematological malignancies. The clinical trial data of our center have good therapeutic effects and are similar to the clinical effects of commercial CAR-T products [14]. However, some patients are not sensitive to CAR-T therapy, and for those who are effective with CAR-T therapy, studies have found that 50% of patients experience recurrence eventually. In this retrospective study, we analyzed patients’ clinical characteristics, baseline hematological parameters and the proportion of T cell subsets isolated from peripheral blood before treatment to identify independent risk factors affecting the efficacy of CAR-T cell therapy. Combined with univariate and multivariate analyses, it was found that the proportions of Tn and Tcm in patients’ peripheral blood CD8 + T cells were positively correlated with clinical outcome after CAR-T treatment. Therefore, we established an early prediction model to analyze the possibility of CR and PR in lymphoma patients based on the proportions of Tcm in Tc and Tn in Tc. This model helps to predict early treatment response in patients with r/r B-NHL. Based on this model, identification of T cell phenotypes prior to CAR-T manufacturing may lead to early intervention by enriching dominant cell subsets or consuming undesirable cell populations or functional states during manufacturing, thereby reducing the failure and relapse rates of CAR-T therapy in r/r NHL.

With the advancement of CAR-T treatment technology, the target population for its treatment is constantly expanding. CAR-T treatment has moved from being considered a third-line treatment or greater to a second-line treatment. With the expansion of the target population for CAR-T treatment, there will inevitably be differences in the responses of different populations to CAR-T treatment. If biomarkers can be used to distinguish them, it can help select more suitable target patients or combine other methods to improve the treatment effect for patients with potential poor efficacy.

In this study, the results of univariate analysis showed that the patient’s baseline tumor burden and ECOG score were significantly associated with remission (CR and PR) after CAR-T cell infusion (*p* < 0.05). Our results are basically consistent with other research findings. Vercellino et al. [8] analyzed the characteristics of 116 patients at the time of decision (TD) to use commercial CAR (axicabtagene ciloleucel, *n* = 49; tisagenlecleucel *n* = 67) and at the time of treatment (TT) and found that the risk factors for early progression of TD and TT during treatment were extranodal (EN) site involvement (≥ 2 sites) and lymphoma burden (LDH, TMTV). This reminds us that disease control before administering CAR-T cells is crucial for better response. Some biomarkers mainly choose to exclude patients who have many potential negative factors that may affect the efficacy of CAR-T through a reverse thinking approach. The research team retrospectively analyzed 34 paired samples in a dual-target CAR-T clinical study and found that 38.2% of patients had low expression of NOXA before CAR-T treatment. Patients with low expression of NOXA received CAR-T treatment, and their PFS and OS were significantly reduced compared to those with high expression of NOXA. ORR and CR rates were also affected [15]. Interestingly, the team’s research found that low expression of NOXA can be reversed by HDACi inhibitors. The HDACi inhibitor Chidamide, independently developed in China, can enhance the killing activity of CAR-T against tumor cells by reversing the low expression of NOXA to high expression [15]. The reversible biomarker provides a foundation for subsequent clinical conversion.

The cellular and molecular diversity of CAR-T cell products is an important factor in the efficacy of CD19 CAR-T cell therapy in LBCL. In this study, we found that T cell heterogeneity in blood samples isolated for CAR-T product manufacturing had a profound impact on clinical outcomes. The high proportion of Tn and Tcm in CD8 + T cells was associated with an improved treatment response. This is consistent with Lamure et al. [16] who suggested that Tisa-cel response was associated with expansion of central memory CD8 + T populations, and Locke et al. [17] described results on the ZUMA-1 cohort that high percentages of naïve-like (CCR7 + CD45RA +) T cell were associated with a higher response rate. Deng et al. [18] found that product cells transfused into patients with durable CR at 3 months were rich in CD8 memory T cell phenotypes compared to PR/PD patients. And Zhang et al. [19] found that knocking out BATF shifts the population toward a more central memory subset and enhances the antitumor activity of CAR-T cells against solid tumors. CD19 CAR-T cells from completely responding individuals with chronic lymphocytic leukemia were enriched in memory-related genes, whereas CAR-T cells from non-responders upregulated effector- and exhaustion-related genes, suggesting that the quantity of Tcm cells is one of the major parameters for the persistence and activity of CAR-T cells [20, 21]. In addition, Lamure et al. [16] found that an increase in Treg cells appeared to be associated with non-response to treatment and could drive clinical relapse. In our study, the proportion of Treg cells was indeed negatively correlated with clinical outcome, although no statistically significant difference was found in the multivariate analysis, which was speculated to be due to the small sample size included, and further clarification is needed in larger cohort studies. This is similar to the finding for the CD4/CD8 ratio. Some investigators believe that the CD8 + T cell-mediated immune response to transgenic CAR-T cells after CAR-T cell infusion limits the persistence of CAR-T cells and increases the risk of relapse in some patients [22]. This study found that the proportion of CD4 + T cells was slightly higher than that of CD8 + T cells in the remission groups. Although this difference was not statistically significant, it also shows that the CD4/CD8 ratio may have an impact on clinical outcome. However, this study has some limitations. First, the number of patients is relatively small, and the predictive model we constructed needs to be further validated in larger prospective studies. In addition, most patients were discharged within 1–3 months after CAR-T treatment, and we only observed short-term efficacy after CAR-T treatment, not long-term outcomes. Articles indicate a positive correlation between the Tn or Tcm ratio and the persistence of CAR-T cells in vivo. There is a high proportion of Tn and memory T cell populations in the patient cohort with CAR-T cells persisting for more than 6 months and sustained remission [23]. We analyzed that our training cohort lasted for 3 years, so there was no difference in the observed results of PFS. Meanwhile, our study is limited to blood, and future studies and analyses of lymph node biopsy should be included to expand the content of this prediction model to comprehensively and deeply analyze the clinical outcomes of patients.

### Supplementary Information

Below is the link to the electronic supplementary material.

Supplementary file1 (TIF 415 kb)

Supplementary file2 (TIF 547 kb)

Supplementary file3 (DOCX 14 kb)

Supplementary file4 (DOCX 17 kb)

## Data Availability

The data that support the findings of this study are available on request from the corresponding author.

## Abbreviations

AUC: Area under the curve
CAR-T: Chimeric antigen receptor T cell
CR: Complete remission
FDA: Food and Drug Administration
NR: No response
ORR: Overall response rates
PBMC: Peripheral blood mononuclear cells
PD: Progressive disease
PR: Partial remission
r/r B-NHL: Refractory or relapsed B cell non-Hodgkin’s lymphoma
ROC: Receiver operating characteristic
SD: Stable disease
Tc: Cytotoxic T or CD8 + T cells
Tcm: Central memory T cell
Tn: Naïve T cell
