# Supplementary figures and images for: A CAR-T response prediction model for r/r B-NHL patients based on a T cell subset nomogram
Source: Cancer Immunol Immunother. 2024 Jan 27;73(2):33. doi: 10.1007/s00262-023-03618-w (PMC10821965; doi:10.1007/s00262-023-03618-w)

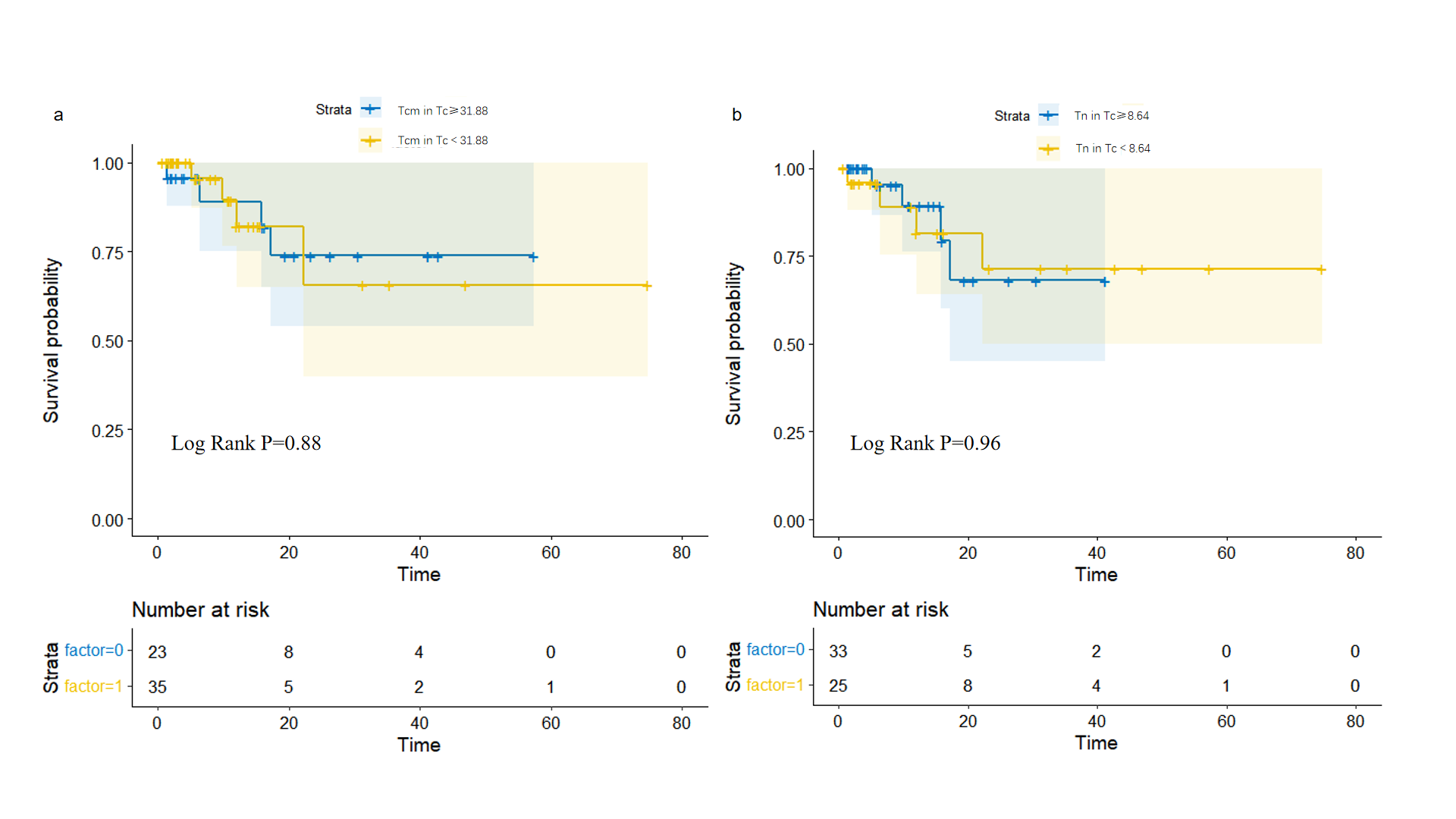

Supplement: Supplementary file 1 — Supplementary file1 (TIF 415 kb) [file 262_2023_3618_MOESM1_ESM.tif]

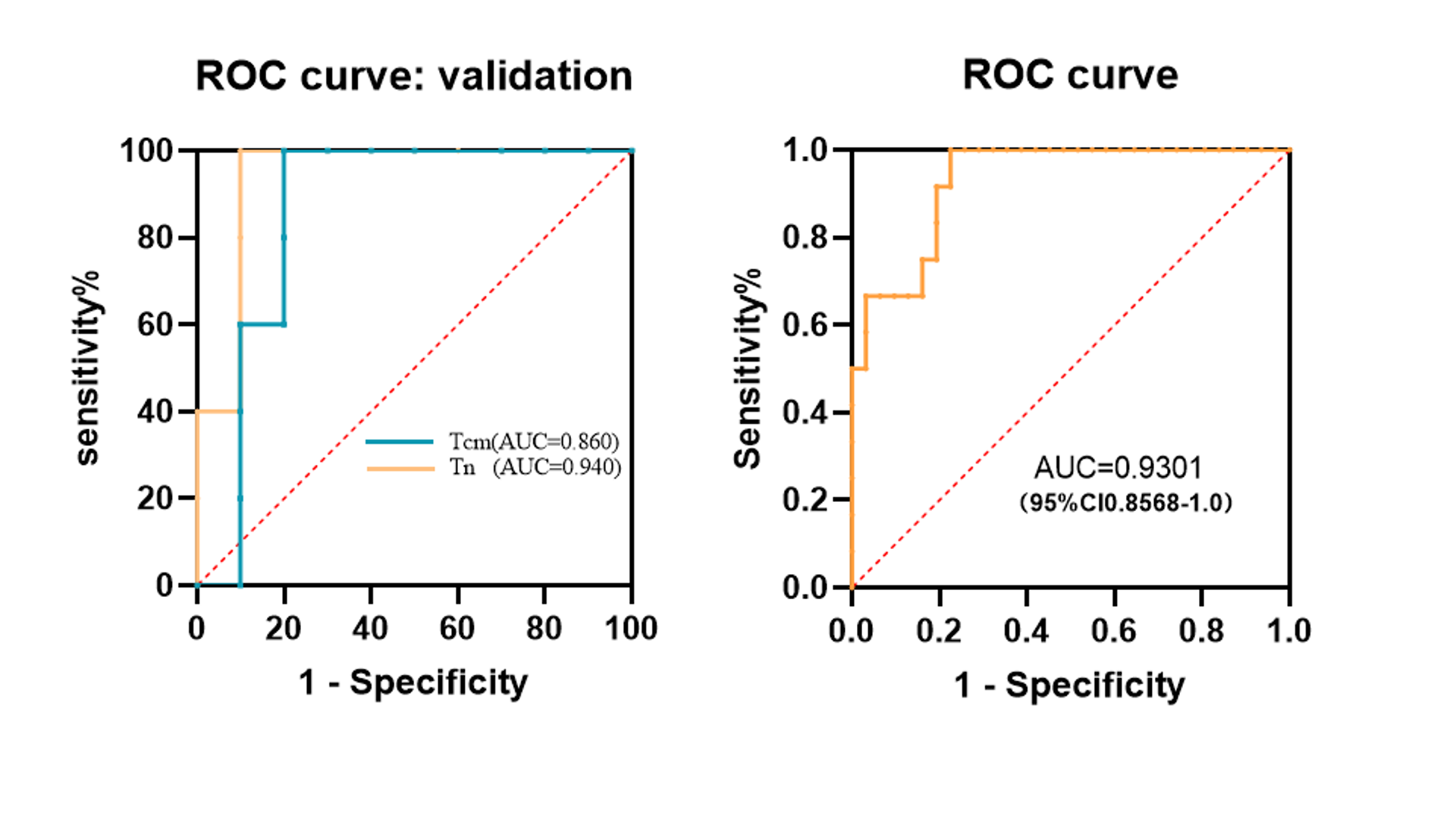

Supplement: Supplementary file 2 — Supplementary file2 (TIF 547 kb) [file 262_2023_3618_MOESM2_ESM.tif]
