# Supplementary material for: A CAR-T response prediction model for r/r B-NHL patients based on a T cell subset nomogram
Source: Cancer Immunol Immunother. 2024 Jan 27;73(2):33. doi: 10.1007/s00262-023-03618-w (PMC10821965; doi:10.1007/s00262-023-03618-w)
Supplement: Supplementary file 3 — Supplementary file3 (DOCX 14 kb) [file 262_2023_3618_MOESM3_ESM.docx]

| **Table1 patient covariates(N=15)** | |
| --- | --- |
| **Covariates** | **n^*^** |
| Age，median(range) | 46（28-67） |
| Gender， n(%) |  |
| Male | 7（46.7）^*^ |
| Female | 8（53.3）^*^ |
| Disease |  |
| DLBCL | 14（93.3）^*^ |
| FL | 1（6.7）^*^ |
| Tumor burden prior CAR-T |  |
| Low | 6（40）^*^ |
| High | 9（60）^*^ |
| Ann Arbor stage |  |
| I-II | 0^*^ |
| III-IV | 15（100）^*^ |
| ECOG score |  |
| ＜2 | 10（66.7）^*^ |
| ≥2 | 5（33.3）^*^ |
| Lines of prior therapies, median(range) | 3（1-6） |
| Bone marrow infiltration |  |
| Yes | 10（23.3）^*^ |
| No | 33（76.7）^*^ |
| Baseline blood count, median(range) |  |
| LDH | 225.3（116.4-7477.8） |
| CRP | 4.52（0.54-341.19） |
| Ferritin | 379（34.3-2222） |
| WBC（×10^9/L） | 3.97(1.48-13.85) |
| Hemoglobin（g/dL） | 109(59-145) |
| Platelet（×10^9/L） | 154(23-670) |
| Baseline T cell subset proportions，median（range） |  |
| CD4/CD8 | 1.1（0.15-13.43） |
| Treg（%） | 5.71（0.07-31.47） |
| Tcm in Th（%） | 7.68（0.38-62.95） |
| Tcm in Tc（%） | 31.88（3.17-64.77） |
| Tn in Th（%） | 13.55（1.15-46.77） |
| Tn in Tc（%） | 8.64（0-48.68） |
| Teff in Th（%） | 2.88（0.16-50.38） |
| Teff in Tc（%） | 33.43（0-79.28） |
| Tem in Th（%） | 34.11（5.72-92） |
| Tem in Tc（%） | 35.12（0-84.97） |
| CAR-T cell dose（×10^6/kg） | 3.82(0.85-12.79) |
| Outcome variable |  |
| CR/PR | 31（72.1）^*^ |
| No response | 12（27.9）^*^ |
| （）^*^, percentage; DLBCL, diffuse large B cell lymphoma; MCL, mantle cell lymphoma; FL, follicular lymphoma; B-LBL, B cell lympho-blastic lymphoma; ECOG, eastern cooperative oncology group; LDH, lactate dehydrogenase; CRP, C-reaction protein; WBC, white blood cells | |
