# Supplementary material for: A CAR-T response prediction model for r/r B-NHL patients based on a T cell subset nomogram
Source: Cancer Immunol Immunother. 2024 Jan 27;73(2):33. doi: 10.1007/s00262-023-03618-w (PMC10821965; doi:10.1007/s00262-023-03618-w)
Supplement: Supplementary file 4 — Supplementary file4 (DOCX 17 kb) [file 262_2023_3618_MOESM4_ESM.docx]

| **Table 2 Univariate logistic regression analyses of the baseline information of CAR-T therapy and CAR-T cell associated with CR or PR** | | | | |
| --- | --- | --- | --- | --- |
| **Variable** | **Remission^*^** | **No-remission^*^** | **Z** | **P** |
| CD4/CD8 | 1.1（0.55，1.78） | 1.14（0.4225，1.4675） | -0.081 | 0.947 |
| Treg | 4.99（1.05，6.84） | 9.225（5.9875，18.76） | -3.222 | 0.001 |
| Tcm in Th | 7.68（4.68，16.67） | 8.155（2.535，15.1525） | -0.596 | 0.565 |
| Tcm in Tc | 36.18（29.16，50.05） | 16.67（7.1575，28.4775） | -3.574 | 0.000 |
| Tn in Th | 15.28（8.59，28） | 8.07（5.8775，18.4125） | -1.814 | 0.071 |
| Tn in Tc | 12.77（7.01，21.34） | 3.275（0.9625，6.3225） | -3.385 | 0.000 |
| Tem in Th | 34.11（24.49，49.9） | 29.985（8.6175，51.13） | -1.164 | 0.254 |
| Tem in Tc | 40.19（21.6，59.47） | 33.765（22.6，47.69） | -0.839 | 0.414 |
| Teff in Th | 2.84（1.4，7.09） | 3.175（1.59，6.5975） | -0.271 | 0.8 |
| Teff in Tc | 33.43（18.47，52.72） | 34.245（17.12，46.315） | -0.460 | 0.659 |
| CAR-T cell dose（×10^6/kg） | 4（2.05，6.65） | 3.65（1.58，6.12） | -1.090 | 0.287 |
| （）inter-quartile range; ^*^Remission includes CR and PR, no-remission refers to NR | | | | |

| **Table 3 Multiple logistic regression analysis of the variables of clinical characteristics of r/r NHL patients and CAR-T cell associated with CR or PR** | | | | | |
| --- | --- | --- | --- | --- | --- |
| **Variable** | **B** | **Wald** | **P** | **OR** | **95CI%** |
| Tumor burden  (Low=1；High=0) | -1.155 | 0.331 | 0.565 | 0.315 | 0.006-16.139 |
| ECOG  (＜2=1；≥2=0) | -3.116 | 3.667 | 0.056 | 0.044 | 0.002-1.076 |
| Treg  (＜5.71=1；≥5.71=0) | -1.560 | 1.583 | 0.208 | 0.210 | 0.018-2.388 |
| Tcm in Tc  (＜31.88=1；≥31.88=0) | 2.934 | 3.942 | 0.047 | 18.794 | 1.038-340.137 |
| Tn in Tc  (＜8.64=1；≥8.64=0) | 2.777 | 4.252 | 0.039 | 16.075 | 1.147-225.226 |

| **Table 4 Area under the ROC curve of Tcm and Tn in Tc for predicting the outcome of lymphoma patient** | | | | |
| --- | --- | --- | --- | --- |
| **Variable** | **AUC** | **Standard error** | **P** | **95%CI** |
| Tcm in Tc | 0.855 | 0.060 | 0.000 | 0.737-0.973 |
| Tn in Tc | 0.836 | 0.063 | 0.001 | 0.712-0.960 |
